# High prevalence of *de novo* metabolic dysfunction-associated fatty liver disease after liver transplantation and the role of controlled attenuation parameter

**DOI:** 10.1186/s12876-023-02940-y

**Published:** 2023-09-12

**Authors:** Lung-Yi Mak, Albert CY Chan, Tiffany CL Wong, Wing-Chiu Dai, Wong-Hoi She, Ka-Wing Ma, Sui-Ling Sin, Ka-Wan Chu, Wai-Kay Seto, Man-Fung Yuen, Chung-Mau Lo, James Fung

**Affiliations:** 1 Department of Medicine, The University of Hong Kong Queen Mary Hospital, 102 Pokfulam Road, Pok Fu Lam, Hong Kong; 2 State Key Laboratory Research of Liver Research, The University of Hong Kong, Pok Fu Lam, Hong Kong

**Keywords:** NAFLD, MAFLD, VCTE, CAP, Liver transplantation, Liver biopsy, Metabolic syndrome, Body mass index

## Abstract

**Background & Aims:**

Although non-alcoholic fatty liver disease (NAFLD) remains an uncommon indication for liver transplantation (LT) in the Chinese, the prevalence of NAFLD is increasing. We aimed to determine the prevalence of *de novo* steatosis and metabolic dysfunction-associated fatty liver disease (MAFLD) after LT.

**Methods:**

Transient elastography assessment for liver stiffness and controlled attenuation parameter (CAP) were performed after LT in 549 patients at median time of 77 months from LT. CAP was compared with implant liver biopsy, and also validated in 42 patients with post-LT liver biopsy. Longitudinal history including diabetes mellitus (DM), dyslipidemia, hypertension, and immunosuppressive regimen were recorded.

**Results:**

The optimal cut-off level of CAP for diagnosing at least mild (≥ S1) and moderate-to-severe steatosis (≥ S2/3) was 266 and 293 dB/m respectively, with AUROC of 0.740 and 0.954 respectively. Using this newly derived cut-off, 28.9% patients have *de novo* NAFLD, of which 95.6% fulfilled the criteria for MAFLD. After multivariate analysis, BMI (HR 1.34), DM (HR 2.01), hypertension (HR 2.03), HDL-cholesterol (HR 0.25), LDL-cholesterol (HR 1.5) and cryptogenic cirrhosis (HR 4.85) were associated with the development of S2/3 graft steatosis. *de novo* NAFLD was associated with higher incidence of new-onset hypertension (p < 0.001), graft dysfunction (defined as ALT > 40 U/L; p = 0.008), but not associated with graft fibrosis (defined as liver stiffness > 12 kPa; p = 0.761).

**Conclusion:**

Although NAFLD remains an uncommon primary liver disease indication for LT in Chinese patients, post-transplant *de novo* graft steatosis is common and the majority is classified as MAFLD. Development of graft steatosis is not associated with an increase in graft fibrosis but was associated with worse metabolic control and graft dysfunction. Routine CAP measurement to detect *de novo* graft steatosis should be considered after LT regardless of the primary indication of LT.

**Supplementary Information:**

The online version contains supplementary material available at 10.1186/s12876-023-02940-y.

## Introduction

Survival after liver transplant (LT) has improved significantly with the availability of potent antiviral therapies and effective immunosuppressive regimens, with an expected 10-year survival of over 80% [1]. Non-liver conditions such as metabolic syndrome, including diabetes mellitus (DM), hypertension, and dyslipidemia, have become important contributors to post-transplant morbidity and mortality. Risk factors for post-transplant metabolic syndrome include immunosuppression, which may favor the development of hyperglycemia, dyslipidemia, and hypertension [2]. Moreover, there is often increase in body weight after surgery, with obesity developing in approximately a third of patients within a few years after transplant [3, 4]. It is therefore not surprising that metabolic syndrome is common after LT, with reported rates of up to 50–60% [5].

The presence of metabolic syndrome has been implicated in the development of recurrent or *de novo* graft non-alcoholic fatty liver disease (NAFLD) [6, 7]. NAFLD after LT may contribute to liver enzyme derangements, leading to graft dysfunction and fibrosis. Previous studies have reported a post-transplant NAFLD recurrence rate ranging from 20 to 40% [8, 9]. This wide variation is largely due to the different diagnostic methods, with most studies relying on retrospective reviews of liver biopsies. As screening all patients using liver biopsy would not be feasible, the true prevalence of graft NAFLD after LT remains unclear. Transient elastography with controlled attenuation parameter (CAP) has been shown to be a rapid, reliable, and repeatable non-invasive method for assessment of liver steatosis with high patient acceptance [10]. Previous studies have shown a strong correlation between CAP and fat accumulation in the liver in a variety of chronic liver diseases [11]. Although CAP has been studied for the assessment of donor steatosis in living donor (LD)LT [12, 13], there is very limited data in liver transplant recipients [14]. By using a non-invasive technique, it is now feasible to screen a large population of liver transplant recipients for the presence of graft steatosis and fibrosis.

Recently, the term ‘metabolic dysfunction-associated fatty liver disease’ (MAFLD) is advocated for patients with excess hepatic steatosis associated with overweight/obesity, presence of metabolic dysregulation, and type 2 DM [15]. MAFLD denotes individuals who are metabolically unhealthy and are at risk of advanced liver disease and cardiovascular complications. In this study, we aimed to validate the use of CAP measurement in post-transplant graft steatosis, and determine the prevalence of *de novo* graft steatosis and MAFLD after LT in a large cohort of post-transplant Chinese patients.

## Patients and methods

Consecutive patients with age 18 years or over undergoing LT from January 2003 to December 2014 at The Liver Transplant Center, Queen Mary Hospital, Hong Kong, were eligible for inclusion in this study (detailed inclusion/ exclusion criteria are shown in the Supplementary appendix). No organs from executed prisoners were used. Patient records were reviewed in detail. The diagnosis of DM, hypertension, and dyslipidemia was established by the use of anti-diabetic agents, anti-hypertensives, and lipid-lowering agents respectively for at least 3 months. The diagnosis was confirmed by review of the clinical notes of all patients. The time of onset of these conditions was estimated by the starting date of the respective medications. Body mass index (BMI) was recorded at the time of LT and time of transient elastography. The diagnosis of MAFLD follows the positive diagnostic criteria in the expert consensus statement, which identify subjects with excess hepatic steatosis to have MAFLD if they have also one of the following 3 criteria: (1) overweight or obese (BMI ≥ 23 kg/m^2^ in Asians), (2) for those with normal BMI: presence of ≥ 2 metabolic risk factors [waist circumference ≥ 90/80 cm in Asian men and women, elevated blood pressure ≥ 130/85, raised plasma triglyceride ≥ 1.7 mmol/L or on treatment, reduced high-density lipoprotein (HDL) cholesterol < 1.0 or 1.3 mmol/L for men or women or on treatment, presence of prediabetes, homeostasis model assessment of insulin resistance score ≥ 2.5, or raised plasma high-sensitivity C-reactive protein > 2 mg/L], or (3) presence of type 2 DM [15]. The present study was approved by the Institutional Review Board/ Ethics Committee of the University of Hong Kong and the Hong Kong West Cluster of Hospital Authority. Written informed consent was obtained from all study subjects prior to any study-related procedures.

### Immunosuppression

All patients received basiliximab as induction therapy, and maintained on long term tacrolimus. A tacrolimus concentration of 8–10 ng/ml was targeted for in the initial 3 months with or without mycophenolate mofetil, followed by 5 to 8 ng/mL thereafter. Additional or alternative immunosuppression including cyclosporine, sirolimus, everolimus, and corticosteroid was used for patients who required additional immunosuppression, or were unable to tolerate tacrolimus at the desired dosage.

### Transient elastography examination

All patients routinely underwent liver stiffness measurement (LSM) and CAP score determination using Fibroscan (Echosens, Paris, France) at various time points after LT. This procedure has been well described previously [16]. All patients were fasted for at least 8 h prior to the procedure. Standard quality criteria were applied for transient elastography. The scan was considered valid only if 10 valid scans were obtained with an interquartile range (IQR) to LSM ratio of < 0.30. The median liver stiffness (LS) of the valid scans was used as the representative value, measured in kilopascals (kPa). CAP was only considered valid with an IQR of < 40 dB/m [17].

### Liver biopsies

Liver biopsy was routinely performed at the time of transplantation for all recipients. Liver steatosis was defined as minimal (< 5%), mild (5–33%), moderate (34–66%), and severe (≥ 67%) on microscopic examination. Routine protocol biopsies were not performed during follow-up, and therefore post-LT biopsies were performed only when clinically indicated to determine the cause of underlying graft dysfunction.

### Statistical analyses

All statistical analyses were performed using SPSS version 23.0 (IBM Corp., Armonk, NY). Chi-squared test was used for categorical variables. Continuous variables were presented as median values and IQR shown in brackets, after confirming non-normal distribution by Kolmogorov Smirnov test (Supplementary appendix). Those variables with skewed distribution were analyzed using Mann-Whitney test. Those with three or more variables were analyzed using the Kruskal-Wallis test. Comparison of two variables with calculation of correlation co-efficient was performed using Pearson method. The Kaplan-Meier method was used to calculate the cumulative incidence of new onset metabolic disorders. The area under receiver operating characteristic (AUROC) curve was used to derive the optimal cut-off of CAP score in predicting ≥ S1, ≥S2, and S3 steatosis using the highest calculated Youden index. Multivariate analysis using binary logistic regression was adopted to determine independent factors associated to S2/3 steatosis. A two-sided P-value of < 0.05 was considered statistically significant.

## Results

**Fig. 1:**
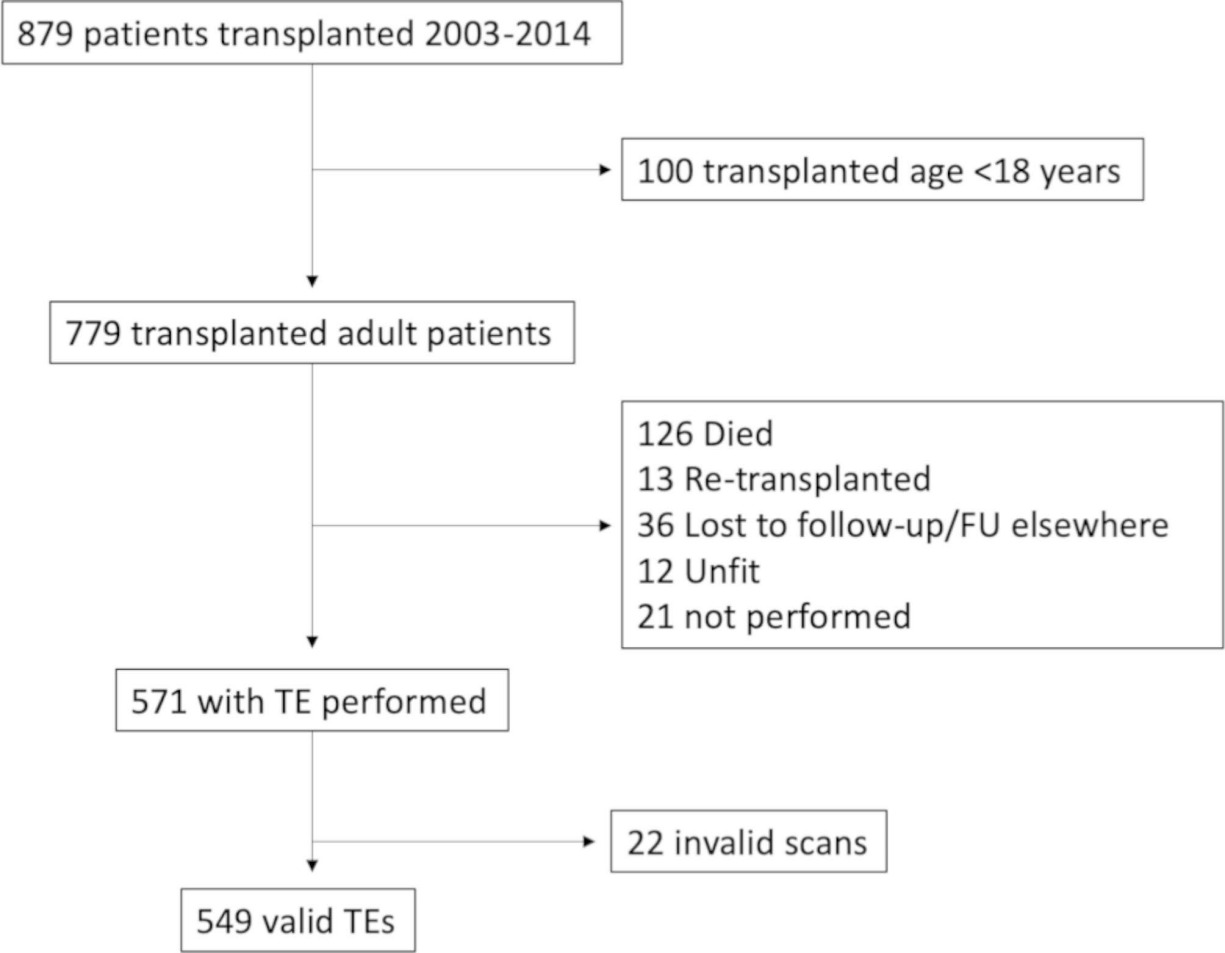
Patient disposition

A total of 879 patients underwent LT from January 2003 to December 2014. Of these, 100 patients were transplanted before the age of 18 years, and were excluded. Of the remaining 779 patients, 571 had transient elastography performed. The remaining 208 patients did not have liver stiffness or CAP measurement because of death, re-transplantation, suboptimal medical conditions, or were lost to follow up (Fig. 1). Twenty-two had invalid scans, resulting in 549 patients with valid measurements (Table 1). The median scan age was 59 (range, 23–78) years, with a median scan time from LT of 77 (range, 6–166) months.

**Table 1:** Baseline parameters of patients with valid transient elastography after liver transplantation

Parameter	Value
Total valid scans (n) Scan age (years) Scan time from transplant (months) Male sex [n(%)] Transplant age (years) Body mass index at transplant (kg/m^2^) Body mass index at scan (kg/m^2^) ***Transplant indication*** Hepatitis B-related Hepatitis C-related Cryptogenic cirrhosis ***Donor type*** Deceased donor Living donor	549 59 (23–78) 77 (6-166) 395 (71.9%) 52 (19–73) 21.3 (13.2–35.8) 23.9 (15.0-39.4) 408 (74.3%) 36 (6.6%) 16 (2.9%) 260 (47.4%) 289 (52.6%)

Continuous variables expressed as median vales (range)

### Prevalence of metabolic factors

At the time of LT, 114 (14.6%), 71 (9.1%), and 17 (2.2%) had established DM, hypertension, and dyslipidemia respectively. After LT, 192 patients developed new onset DM, with cumulative rate of 17.9%, 24.0%, 32.3%, and 38.4% at 1, 5, 10, and 15 years respectively, 389 patients had new onset hypertension, with cumulative rate of 23.8%, 44.4%, 65.3%, and 72.5% at 1, 5, 10, and 15 years respectively, and 164 patients developed new onset dyslipidemia, with cumulative rate of 2.5%, 14.4%, 24.7%, and 36.6% at 1, 5, 10, and 15 years respectively.

### Correlation of CAP score with implantation biopsy

**Fig. 2:**
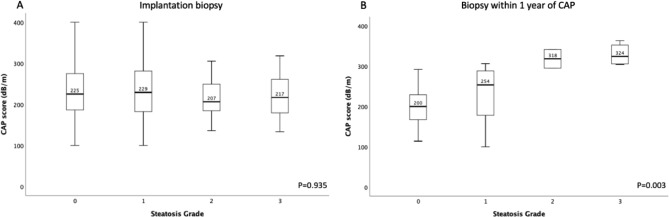
Median CAP scores according to steatosis grade at (**A**) the time of liver implantation and (**B**) liver biopsy within 1 year of CAP measurement

Of the 549 patients with valid transient elastography performed, 422 (76.9%) had < 5% steatosis, 101 (18.4%) had 5–33% steatosis, 18 (3.3%) had 33–66% steatosis, and 8 (1.4%) had > 66% steatosis from the liver biopsy taken at the time of implantation. There was no significant difference in post-transplant CAP scores when correlating with the degree of graft steatosis at the time of transplant. For patients with < 5%, 5–33%, 33–66%, and > 66% steatosis at implant biopsy, the median CAP scores were 225 (range, 114–400), 229 (range, 121–400), 207 (range, 136–305), and 217 (range, 133–318) dB/m respectively (p = 0.935) (Fig. 2A).

### Correlation of CAP with liver biopsy after liver transplantation

Liver biopsies performed within a year of valid CAP measurements were available for 42 patients. Of these, 22 (52.4%) had minimal (< 5%) steatosis, 14 (33.3%) had mild (5–33%) steatosis, 2 (4.8%) had moderate (34–66%) steatosis, and 4 (9.5%) had severe (≥ 67%) steatosis. A significantly higher CAP score was observed for higher grades of steatosis, from 200, 254, 318, and 324 dB/m for minimal, mild, moderate, and severe steatosis respectively (p = 0.003) (Fig. 2B). Given the small numbers of those with moderate and severe steatosis, these were grouped together for analysis. The AUROC for diagnosing at least mild, moderate, and severe steatosis using CAP measurement was 0.740, 0.954, and 0.951 respectively (Supplementary Table 1), with an optimal cut-off of 266, 293, and 301 dB/m respectively (Supplementary Fig. 1A, B and C).

### Prevalence and risk factors for moderate-severe steatosis and MAFLD

Using the optimal CAP score cut-offs as derived, the prevalence of at least mild (≥ S1), moderate (≥ S2), and severe steatosis (S3) was 28.9%, 17.1%, and 14.0% respectively in the 549 patients with CAP measurements. Of the 159 patients with NAFLD, 152 (95.6%) fulfilled the criteria for MAFLD.

A total of 94 patients had moderate-severe (S2/3) steatosis. No difference was observed in the diagnosis of HBV, HCV, or alcoholic liver disease between those with and without S/3 steatosis, with a higher rate of cryptogenic cirrhosis (7.4% vs. 2.0% respectively, p = 0.011). Ninety-three (98.9%) patients had MAFLD, of which 88 (94.6%) had overweight/obesity, 46 (49.5%) had type 2 DM, and 33 (35.5%) had at least 2 metabolic risk abnormalities. Only 1 patient did not fulfil the MAFLD criteria with standalone hypertension, and interestingly, this patient had intermittent alcohol intake post transplantation for hepatitis B-related cirrhosis.

Patients with S2/3 steatosis, when compared to S0/1 steatosis, had higher cumulative rates of DM (54.3% vs. 35.2% respectively, p = 0.001), hypertension (85.1% vs. 60.2% respectively, p < 0.001), and dyslipidemia (39.4% vs. 23.7% respectively, p = 0.002). The incidence of new-onset hypertension was also higher among those with S2/3 steatosis compared to those without (70.2% vs. 48.6%, p < 0.001), while similar incidence rates for new-onset DM and dyslipidaemia were observed (Supplementary Fig. 2). A higher BMI at the time of transplant and at CAP measurement was observed for those with S2/3 steatosis (23.5 kg/m^2^ vs. 20.7 kg/m^2^, p < 0.001 and 26.6 kg/m^2^ vs. 23.1 kg/m^2^, p < 0.001 respectively). There was an increasing rate of moderate-severe steatosis observed for those with BMI < 18.5, 18.5–22.9, 23.0-24.9, 25-29.9, and ≥ 30 kg/m^2^ (0%, 3.1%, 10.0%, 36.0%, and 48.6% respectively, p < 0.001, Supplementary Fig. 3).

Patients with S2/3 steatosis, compared to those without, had higher fasting glucose (6.3 vs. 5.4 mmol/L respectively, p < 0.001), low density lipoprotein (LDL)-cholesterol (2.6 vs. 2.2 mmol/L respectively, p < 0.001), triglyceride (1.5 vs. 1.0 mmol/L respectively, p < 0.001), and lower HDL-cholesterol 1.1 vs. 1.3 mmol/L respectively, p < 0.001) at the time of transient elastography. The results are summarized in Supplementary Table 2.

After multivariate analysis, only BMI at the time of CAP measurement (and not at the time of transplant), cryptogenic cirrhosis as a primary liver disease, the presence of DM, hypertension, HDL-cholesterol and LDL-cholesterol levels remained significant factors associated with S2/3 steatosis (Table 2). The degree of donor graft steatosis at the time of implant (i.e. implant biopsy) was also not associated with S2/3 steatosis (Supplementary Table 4).

**Table 2:** Multivariate analysis of factors associated with S2/3 graft steatosis

Parameter	HR (95% CI)	P value
BMI at CAP measurement Diabetes mellitus Hypertension HDL-cholesterol LDL-cholesterol Cryptogenic cirrhosis	1.34 (1.20–1.49) 2.01 (1.09–3.71) 2.03 (1.04–3.94) 0.25 (0.10–0.58) 1.50 (1.05–2.16) 4.85 (1.45–16.3)	< 0.001 0.026 0.037 0.001 0.027 0.011

### Steatosis and graft dysfunction

Patients with moderate-severe steatosis, compared to those without, had higher alanine aminotransferase (ALT) (28 vs. 23 U/L respectively, p < 0.001), and a higher rate of graft dysfunction, defined as serum ALT > 40 U/L (25.5% vs. 14.1%, p = 0.008; Supplementary Fig. 2). There was no significant difference between the 2 groups with respect to bilirubin, alkaline phosphatase (ALP), aminotransferase aspartate, and gamma glutamyl transpeptidase (GGT) levels (p = 0.964, p = 0.559, p = 0.461, and p = 0.066 respectively. The higher prevalence of biliary disease/complications and cholestasis observed in post-LT settings may have masked the difference in GGT levels. By excluding patients with elevated ALP, a significantly higher GGT level was able to be observed for those with S2/3 steatosis (39 vs. 28 IU/L, p = 0.003). Using the local laboratory cut offs of 58 and 45 U/L for males and females respectively, 16 patients (all with MAFLD) had elevated ALT at the time of CAP measurement, with a median level of 62 U/L (range, 57–143). Seven patients had liver biopsies within 1 year, all with steatosis and 3 with evidence of steatohepatitis.

### Steatosis and graft fibrosis

There was no significant correlation between CAP and liver stiffness scores (p = 0.886), and no significant difference in the median liver stiffness between those with S2/3 and S0/1 steatosis (5.4 kPa vs. 4.9 kPa, p = 0.091). Of the 94 patients with moderate-severe steatosis, 4 (4.3%) had liver stiffness ≥ 12 kPa, compared to 3.5% among those with minimal or mild graft steatosis (p = 0.761; Supplementary Fig. 2). The characteristics of these 4 patients (all with MAFLD) are summarized in Supplementary Table 3. One patient had steatosis with bridging fibrosis with liver stiffness of 14.3 kPa, who was transplanted for acute flare of chronic hepatitis B, remained negative for serum hepatitis B surface antigen (HBsAg) and hepatitis B virus DNA after transplant without any episodes of rejection.

## Discussion

Graft steatosis after LT can occur as a recurrence of underlying disease for those transplanted for NASH-related cirrhosis, or as *de novo* NAFLD. As protocol liver biopsies are no longer routinely performed, large cohort screening for post-transplant steatosis using liver biopsy is not feasible. Although non-invasive assessment of hepatic steatosis using CAP measurement has been validated previously, there is a paucity of data regarding its use in post-transplant graft steatosis. Moreover, it is recently reported that the cut-off value of CAP for hepatic steatosis in the recipients of LT is different from the non-transplanted population (270 dB/m instead of 248 dB/m) [18]. The current study established an optimal cutoff for moderate-severe steatosis of 293 dB/m with an AUROC of 0.954 (100% sensitivity, 89% specificity, 60% positive predictive value and 100% negative predictive value), and this cut-off was used to determine the prevalence of moderate-severe steatosis in the post-transplant cohort.

Although NAFLD is common in Hong Kong [19, 20], NASH is a rare indication for LT, highlighted by the fact that none of the LT recipients in the current study had NAFLD coded as a primary liver indication. However, the prevalence of *de novo* graft steatosis was common (28.9%), and over 95% of these had MAFLD. Interestingly, graft status and BMI at the time of transplant were not independent factors associated with S2/3 steatosis, whereas BMI at the time of CAP measurement was. This suggests that the development of MAFLD is dependent on the recipient risk factors, and consistent with a previous study of split liver transplantation where recipient factors were shown to be associated with NASH [21]. In another study of 68 patients, 18% developed *de novo* NAFLD which was associated with weight gain after transplant [22]. In our study, there was significant increase in the median BMI after LT (from 21.3 to 23.9 kg/m^2;^ p < 0.001), which might be able to explain the high rate of *de novo* NAFLD after LT among patients who were transplanted for non-NAFLD related causes. The exact mechanisms for post-LT weight gain in our population were not clear, which were likely multifactorial (e.g., immunosuppressant use, reversal of frailty, improvement in appetite after stabilization of medical condition).

The BMI was a strong independent risk factor for S2/3 steatosis, which was observed in almost half of the patients with BMI ≥ 30 kg/m^2^. The presence of metabolic risk abnormalities (such as DM, hypertension, low HDL-cholesterol, overweight/obesity) was associated with *de novo* S2/3 graft steatosis, highlighting that these are indeed classified as MAFLD according to the recently advocated diagnostic criteria [15]. The current study is the first to report the risk factors and implications of *de novo* MAFLD in the post-LT population, with almost all patients with S2/3 steatosis fulfilling the MAFLD criteria. *de novo* moderate to severe MAFLD was associated with new-onset hypertension, graft dysfunction (Supplementary Fig. 2), and worse metabolic blood profiles (Supplementary Table 2). The rarity of NAFLD as a primary liver disease indication together with < 5% of graft biopsy at implantation showing S2/3 steatosis implicates the intercalated and bidirectional effects of metabolic factors in both contributing to new-onset steatosis after LT, which in turn, is associated with worse metabolic control and graft dysfunction.

The finding of cryptogenic cirrhosis being an independent risk factor for S2/3 graft steatosis did support the long-suspected notion that these could potentially have burnt-out NASH. Since the pre-LT characteristics of those with cryptogenic cirrhosis showed no difference in BMI or prevalence of diabetes, hypertension or dyslipidemia when compared to those with known causes, the true prevalence of burnt-out NASH among patients with cryptogenic cirrhosis remains unclear. Other causes may include prior chronic hepatitis B infection with subsequent HBsAg seroclearance, given that half of the cryptogenic patients were positive for hepatitis B core antibody (data not shown).

Despite the presence of S2/3 steatosis, only a small proportion (3.8%) in the current study had liver stiffness ≥ 12 kPa, suggesting that advance fibrosis/cirrhosis remained rare. This is consistent with the findings of a previous retrospective study of 2360 post-transplant biopsies showing that significant steatosis was not associated with a higher fibrosis stage [23]. The lack of fibrosis may be in part due to the insufficient duration from transplant to liver stiffness measurement (despite a median duration of 77 months), as time is required for both steatosis and subsequent fibrosis to development.

There were several limitations in the current study. Firstly, the diagnosis of steatosis in the large cohort was based on CAP score measurement. However, the CAP score has been shown to correlate well with degrees of steatosis, and the optimal cut-off was determined using available liver histology. It would not have been feasible to perform liver biopsies for all patients in such a large cohort. Moreover, although liver biopsy remains the gold standard, it is also subjected to sampling errors, especially when the fat distribution is not homogenous throughout the liver. Nevertheless, we included subjects with clinically-indicated liver biopsies (i.e. persistently abnormal liver biochemistry) within 1 year of valid transient elastography measurement to ensure the validity of correlation with histology. Secondly, the diagnosis of DM, hypertension, and dyslipidemia were based on treatment received, and this may have underestimated the true prevalence of these metabolic conditions. However, it is likely that those with evidence of these conditions would have been started on appropriate treatment, and therefore would not have been missed. Thirdly, CAP was measured at variable duration after LT and not at fixed timepoints. However, the wide range of post-LT duration for up to 121 months allows for the assessment of steatosis over time. Although steatosis was shown to increase in prevalence with longer duration after transplant [23], this was not observed in the current study. Lastly, some of the metabolic risk parameters such as waist circumference, homoeostasis model assessment of insulin resistance, and plasma high-sensitivity C-reactive protein level were not available. However, it is unlikely to have affected the results as > 95% of NAFLD had fulfilled the MAFLD criteria using available parameters.

In conclusion, although NASH remains an uncommon indication for LT in Chinese patients, post-transplant metabolic disorders are common, and associated with the development of MAFLD in over 25% after liver transplantation. Moderate to severe graft steatosis detected by CAP is associated with new-onset hypertension, graft dysfunction, and worse metabolic profile. Routine CAP measurement should be considered in post-LT patients to identify *de novo* graft steatosis and associated metabolic risk factors, so that closer monitoring and earlier treatment for the metabolic risk factors can be implemented. Long-term follow-up of this cohort will be required to observe whether development of graft steatosis and/or MAFLD is associated with increase in graft fibrosis.

### Electronic supplementary material

Below is the link to the electronic supplementary material.

**Supplementary Table 1**. Accuracy of CAP score in predicting ≥ S1, ≥ S2 and S3 graft steatosis. **Supplementary table 2**. Patients with and without moderate-severe steatosis. Supplementary table 3. Patients with S2/3 steatosis and liver stiffness measurement ≥ 12kPa. **Supplementary Table 4**. Relationship between implant biopsy (i.e. at time of LT, performed for all subjects), post-LT biopsy (performed for clinical indication and within 1 year of TE) and CAP-defined graft steatosis. **Supplementary Figure 1**. Receiver operating characteristic curve of CAP in the prediction of (A) ≥ S1 steatosis (B) ≥ S2/3 steatosis and (C) S3 steatosis. **Supplementary Figure 2**. Significance of moderate to severe graft steatosis on the rate of new-onset metabolic risk factors, graft dysfunction and graft fibrosis. Graft dysfunction was defined as serum alanine aminotransferase > 40 U/L. Graft fibrosis was defined as liver stiffness > 12 kPa. CAP: controlled attenuation parameter; DM: diabetes mellitus; HTN: hypertension; LP: dyslipidaemia. **Supplementary Figure 3**. Prevalence of S2/3 steatosis according to BMI after liver transplantation.

## Data Availability

The datasets used and/or analysed during the current study are available from the corresponding author on reasonable request.

## Abbreviations

ALT: Alanine aminotransferase
ALP: Alkaline phosphatase
AUROC: Area under receiver operating characteristic
HBsAg: Hepatitis B surface antigen
BMI: Body mass index
CAP: Controlled attenuation parameter
DDLT: Deceased donor liver transplantation
DM: Diabetes mellitus
GGT: Gamma glutamyl transpeptidase
HBV: Hepatitis B virus
HCV: Hepatitis C virus
HDL: High-density lipoprotein
IQR: Interquartile range
kPa: Kilopascals
LDL: Low density lipoprotein
LDLT: Living donor liver transplantation
LS: Liver stiffness
LSM: Liver stiffness measurement
LT: Liver transplantation
MAFLD: Metabolic dysfunction-associated fatty liver disease
NAFLD: Non-alcoholic fatty liver disease
NASH: Non-alcoholic steatohepatitis
