# Supplementary material for: High prevalence of de novo metabolic dysfunction-associated fatty liver disease after liver transplantation and the role of controlled attenuation parameter
Source: BMC Gastroenterol. 2023 Sep 12;23:307. doi: 10.1186/s12876-023-02940-y (PMC10498589; doi:10.1186/s12876-023-02940-y)
Supplement: Supplementary file 1 — Supplementary Table 1. Accuracy of CAP score in predicting ≥ S1, ≥ S2 and S3 graft steatosis. Supplementary table 2. Patients with and without moderate-severe steatosis. Supplementary table 3. Patients with S2/3 steatosis and liver stiffness measurement ≥ 12kPa. Supplementary Table 4. Relationship between implant biopsy (i.e. at time of LT, performed for all subjects), post-LT biopsy (performed for clinical indication and within 1 year of TE) and CAP-defined graft steatosis. Supplementary Figure 1. Receiver operating characteristic curve of CAP in the prediction of (A) ≥ S1 steatosis (B) ≥ S2/3 steatosis and (C) S3 steatosis. Supplementary Figure 2. Significance of moderate to severe graft steatosis on the rate of new-onset metabolic risk factors, graft dysfunction and graft fibrosis. Graft dysfunction was defined as serum alanine aminotransferase > 40 U/L. Graft fibrosis was defined as liver stiffness > 12 kPa. CAP: controlled attenuation parameter; DM: diabetes mellitus; HTN: hypertension; LP: dyslipidaemia. Supplementary Figure 3. Prevalence of S2/3 steatosis according to BMI after liver transplantation. [file 12876_2023_2940_MOESM1_ESM.docx]

**High prevalence of *de novo* metabolic dysfunction-associated fatty liver disease after liver transplantation and the role of controlled attenuation parameter**

Lung-Yi Mak, Albert CY Chan, Tiffany CL Wong, Wing-Chiu Dai, Wong-Hoi She, Ka-Wing Ma, Sui-Ling Sin, Ka-Wan Chu, Wai-Kay Seto, Man-Fung Yuen, Chung-Mau Lo, James Fung

**Supplementary Materials**

**Supplementary information for Methods**

**Patients**

Inclusion criteria:

- underwent liver transplantation from January 2003 to December 2014 at the Liver Transplant Centre, Queen Mary Hospital, Hong Kong
- age ≥18 years old at the time of liver transplantation
- had transient elastography measurement after liver transplantation

Exclusion criteria:

- invalid transient elastography measurement
- failed transient elastography measurement
- re-transplanted
- suboptimal medical conditions
- lost to follow up
- death

**Statistical analysis**

Test for normality of data by Kolmogorov Smirnov test

|  | P value | Normal distribution? |
| --- | --- | --- |
| Age at LT | <0.001 | No |
| Age at TE | <0.001 | No |
| BMI at LT | 0.002 | No |
| BMI at TE | 0.004 | No |
| Liver stiffness | <0.001 | No |
| CAP | 0.036 | No |
| ALT | <0.001 | No |
| AST | <0.001 | No |
| ALP | <0.001 | No |
| GGT | <0.001 | No |
| Bilirubin | <0.001 | No |
| Fasting glucose | <0.001 | No |
| Total cholesterol | <0.001 | No |
| HDL cholesterol | <0.001 | No |
| LDL cholesterol | <0.001 | No |
| Triglyceride | <0.001 | No |

Supplementary Table 1. Accuracy of CAP score in predicting ≥S1, ≥S2 and S3 graft steatosis

|  | ≥S1 steatosis | ≥S2 steatosis | S3 steatosis |
| --- | --- | --- | --- |
| AUROC  Optimal cut-off (dB/m)  Sensitivity  Specificity  Positive predictive value  Negative predictive value | 0.740  266  0.65  0.91  0.87  0.74 | 0.954  293  1.00  0.89  0.60  1.00 | 0.951  301  1.00  0.89  0.50  1.00 |

Supplementary table 2. Patients with and without moderate-severe steatosis

| Parameters | No/minimal/mild steatosis  (n=455) | Moderate/severe steatosis  (n=94) | P value |
| --- | --- | --- | --- |
| Male sex (%)  Hepatitis B  Hepatitis C  Alcoholic liver disease  Cryptogenic cirrhosis  At transplant  Age (years)  BMI (kg/m^2^)  Living donor  At CAP measurement  Age (years)  BMI (kg/m^2^)  Time from LT (months)  Diabetes mellitus  Hypertension  Dyslipidemia  *Liver parameters*  Bilirubin  ALP (U/L)  GGT (U/L)  ALT (U/L)  AST (U/L)  Liver stiffness (kPa)  *Fasting levels*  Glucose  Total cholesterol  HDL-cholesterol  LDL-cholesterol  Triglyceride | 71.9%  73.4%  7.0%  3.3%  2.0%  52 (19-73)  20.7 (13.2-34.0)  51.4%  59 (23-77)  23.1 (15.0-38.4)  77 (6-166)  35.2%  60.2%  23.7%  12 (3-65)  79 (31-727)  30 (10-590)  23 (6-203)  25 (12-183)  4.9 (1.6-39.7)  5.4 (3.5-28.0)  4.2 (2.3 -12.4)  1.3 (0.1-11.0)  2.2 (0.3-5.2)  1.1 (0.4-6.5) | 72.3%  78.7%  4.3%  2.1%  7.4%  53 (23-66)  23.5 (16.4-35.8)  58.5%  58 (32-78)  26.6 (20.2-39.4)  72 (15-164)  54.3%  85.1%  39.4%  13 (2-36)  82 (43-232)  39 (10-646)  28 (8-143)  26 (14-66)  5.4 (3.0-19.2)  6.3 (3.3-13.2)  4.4 (2.8-9.7)  1.1 (0.6-2.5)  2.6 (1.0-5.5)  1.5 (0.6-5.1) | 0.926  0.283  0.322  0.750  0.011  0.148  <0.001  0.211  0.257  <0.001  0.889  0.001  <0.001  0.002  0.964  0.559  0.066  <0.001  0.461  0.091  <0.001  0.091  <0.001  <0.001  <0.001 |

Supplementary table 3. Patients with S2/3 steatosis and liver stiffness measurement ≥12kPa

| Patient | LT indication | LS (kPa) | CAP (dB/m) | Biopsy | Remarks |
| --- | --- | --- | --- | --- | --- |
| #1  #2  #3  #4 | HBV cirrhosis  HBV cirrhosis  Severe HBV flare  Cryptogenic cirrhosis/?NASH | 19.2  19.2  14.3  13.0 | 400  369  341  363 | -  -  NASH + bridging fibrosis  NASH + no fibrosis | No HBV recurrence  No HBV recurrence  No HBV recurrence |

CAP: controlled attenuation parameter, HBV: hepatitis B virus, LS: liver stiffness, LT: liver transplant, NASH: non-alcoholic steatohepatitis,

Supplementary Table 4. Relationship between implant biopsy (i.e. at time of LT, performed for all subjects), post-LT biopsy (performed for clinical indication and within 1 year of TE) and CAP-defined graft steatosis

|  | | **Implant biopsy** | | | |
| --- | --- | --- | --- | --- | --- |
|  |  | No steatosis (<5% fat) | Mild steatosis (5 to <33%) | Moderate steatosis (33 to <66%) | Severe steatosis (≥66%) |
| **Post-LT liver biopsy within 1 year of TE** | No steatosis (<5% fat) | 15 | 7 | 0 | 0 |
|  | Mild steatosis (5 to <33%) | 8 | 4 | 2 | 0 |
|  | Moderate steatosis (33 to <66%) | 1 | 0 | 1 | 0 |
|  | Severe steatosis (≥66%) | 3 | 1 | 0 | 0 |
|  | | | | | |
| **CAP-defined graft steatosis grade** | No steatosis (CAP <266 dB/m) | 301 | 68 | 14 | 6 |
|  | Mild steatosis (CAP 266 -293 dB/m) | 49 | 14 | 1 | 1 |
|  | Moderate steatosis (CAP 293 -301 dB/m) | 11 | 4 | 2 | 0 |
|  | Severe steatosis (CAP >301 dB/m) | 60 | 15 | 1 | 1 |

CAP: controlled attenuation of parameter, LT: liver transplantation, TE: transient elastography

Supplementary Figure 1. Receiver operating characteristic curve of CAP in the prediction of (A) ≥S1 steatosis (B) ≥S2/3 steatosis and (C) S3 steatosis


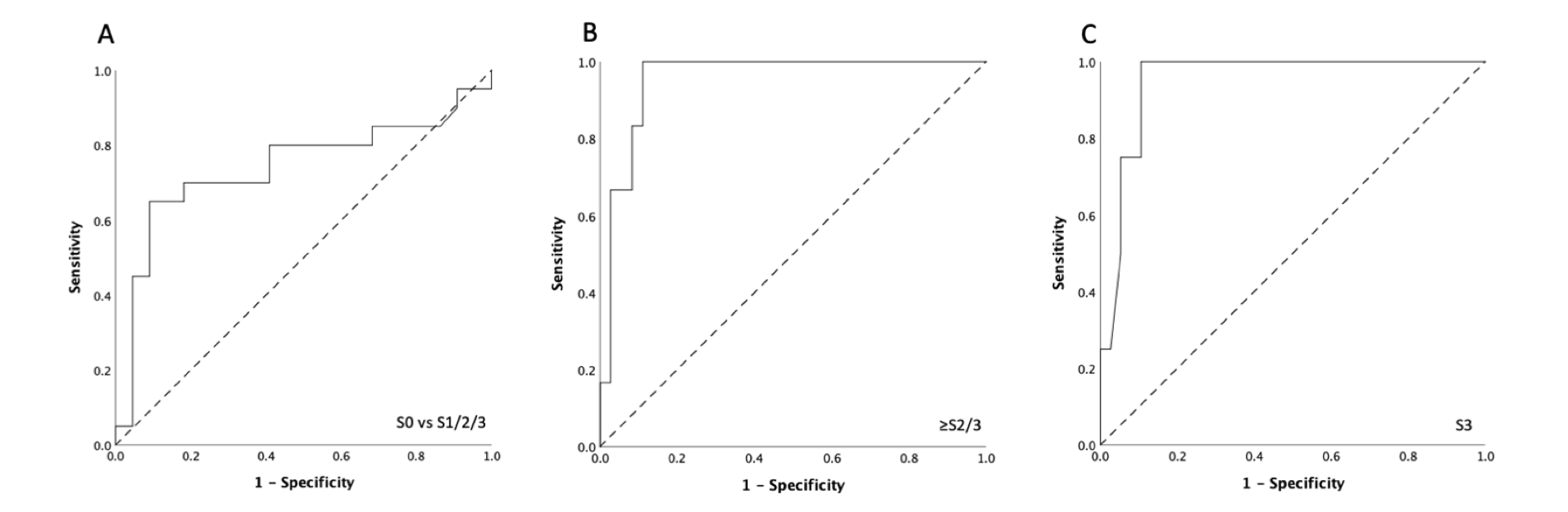


Supplementary Figure 2. Significance of moderate to severe graft steatosis on the rate of new-onset metabolic risk factors, graft dysfunction and graft fibrosis. Graft dysfunction was defined as serum alanine aminotransferase >40 U/L. Graft fibrosis was defined as liver stiffness >12 kPa. CAP: controlled attenuation parameter; DM: diabetes mellitus; HTN: hypertension; LP: dyslipidaemia


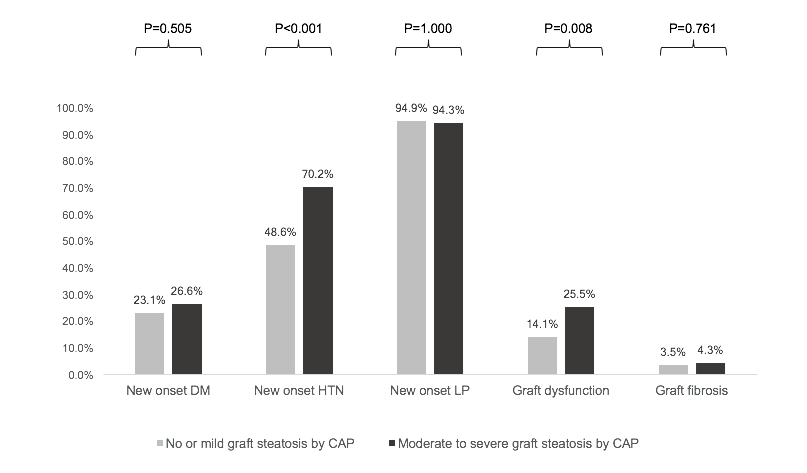


Supplementary Figure 3. Prevalence of S2/3 steatosis according to BMI after liver transplantation
